# Correlation between mild cognitive impairment and flourishing among Chinese residents: a cross-sectional study

**DOI:** 10.3389/fpsyg.2025.1550013

**Published:** 2025-03-26

**Authors:** Hangqin Lv, Xin Yi, Xiangjun Guo, Meichuan Lin, Dingxi Bai, Xingyu Nie, Xue Wang, Xiaoyun Liu

**Affiliations:** ^1^School of Nursing, Chengdu University, Chengdu, China; ^2^School of Nursing, Chengdu University of Traditional Chinese Medicine, Chengdu, China

**Keywords:** mild cognitive impairment, flourishing, correlation analysis, Chinese residents, AD8

## Abstract

**Objective:**

This study aimed to investigate the correlation between mild cognitive impairment and flourishing among Chinese residents.

**Methods:**

A total of 527 community residents aged ≥18 years were recruited from December 2023 to April 2024. Based on the results of the Ascertain Dementia 8-Item Informant Questionnaire (AD8), participants were classified into a healthy group (*n* = 356) and a mild cognitive impairment (MCI) group (*n* = 171). General demographic data, including age, gender, height, weight, place of residence, education level, marital status, household composition, personal income, occupation, and the flourishing scale (FS) were collected for statistical analysis. The analysis was performed using Statistical Product and Service Solutions software. Chi-square test was used to compare differences between the groups, while Kendall’s correlation analysis and multivariate logistic regression were applied to assess the relationship between flourishing and MCI.

**Results:**

Comparisons between the healthy and MCI groups showed that the FS scores in the healthy group were significantly higher than those in the MCI group (*p* < 0.01). Kendall’s correlation analysis revealed that the score of AD8 was negatively correlated with FS (*r* = −0.237, *p* < 0.01). Multivariate analysis indicated that age [odds ratio (OR) = 1.451, 95% confidence interval (CI; 1.107–1.902), *p* = 0.007], place of residence [OR = 5.523, 95% CI (3.572–8.539), *p* < 0.001], and FS [OR = 0.421, 95%CI (0.311–0.569), *p* < 0.001] were correlated with MCI.

**Conclusion:**

Flourishing levels are negatively correlated with MCI, and higher levels of flourishing associated with a lower risk of MCI. This suggests that flourishing may serve as a protective factor against cognitive decline. Additionally, age and place of residence are identified as risk factors for MCI.

## Introduction

Dementia is a major cause of disability in individuals aged >65 years worldwide, including China, and presents significant challenges for policymakers, healthcare providers, and family members (Jia et al., 2020b). The onset and progression of Alzheimer’s disease (AD), the leading cause of dementia, are gradual and span several years to decades before symptoms become evident (Jack et al., 2013; Villemagne et al., 2013). It is characterized by progressively worsening cognitive and functional impairments. Mild cognitive impairment (MCI), considered the first clinical manifestation of AD, occurs when an individual exhibits below-average performance on standardized neuropsychological tests (Studart and Nitrini, 2016). When cognitive impairment becomes significant enough to interfere with daily functioning, the diagnosis of AD is established (Albert et al., 2011). AD is irreversible and can only have its progression delayed. Therefore, paying attention to MCI in the early stages is crucial. Early diagnosis and timely intervention during this phase can help slow down the progression of AD.

MCI imposes a significant burden, affecting the quality of life and psychological well-being (Gates et al., 2014). Those with MCI exhibit lower levels of social support, self-esteem, life satisfaction, positive affect, optimism, and hope but higher levels of negative affect compared with healthy individuals (Dos Santos et al., 2018). Currently, the research on the psychological aspects of MCI is limited. However, some studies indicated that individuals with dementia exhibit a lack of recognition, assessment, and even the ability to feel negative emotions (Balconi et al., 2015; Oliver et al., 2015; Bora et al., 2016) and preserve the ability to recognize positive emotions (Dos Santos et al., 2018; Oliver et al., 2015; Bora et al., 2016; Goodkind et al., 2015; St. Jacques et al., 2015). The incidence of cognitive impairment was also associated with optimism in a study evaluating 4,624 elderly people over 4 years, which showed that high optimism was a protective factor against cognitive impairment and played an important role in maintaining cognitive functioning (Dos Santos et al., 2018; Gawronski et al., 2016). Thus, we should pay more attention to positive psychology. The mission of positive psychology is to understand and foster the factors that allow individuals, communities, and societies to flourish (Fredrickson, 2001).

The flourishing theory, an emerging field of well-being within positive psychology, was originally developed to operationalize mental health in response to the notion that well-being is not merely the absence of mental illness (Jahoda, 1959), but Rather, it encompasses the cultivation of positive emotional experiences, eudaimonic well-being, and meaningful social engagement and contribution (Keyes, 2002; Mock and Smale, 2023). Keyes (2002) conceptualized flourishing within the Mental Health Continuum Model, which defines mental health as a spectrum ranging from languishing (a state of psychological distress and low well-being) to flourishing (optimal mental health with high levels of emotional, psychological, and social well-being). Similarly, Seligman’s (2011) PERMA model identifies five core elements essential for flourishing: Positive Emotion, Engagement, Relationships, Meaning, and Accomplishment, each contributing to overall life satisfaction and psychological resilience.

Most research on flourishing focused on adolescents and the elderly (Parsons et al., 2022; Otgon et al., 2023), its relevance to individuals with MCI remains largely unexplored. Flourishing is the pinnacle of good mental health, and it is constituted by an affective state and psychological and social functioning (Mjøsund, 2021) — elements that have been associated with cognitive health (Rodrigues and Delerue-Matos, 2025; Yang et al., 2024) —understanding its relationship with MCI may provide insights into potential protective mechanisms against cognitive decline. Consequently, this study aims to explore the correlation between MCI and flourishing among Chinese residents.

## Methods

### Study design

This cross-sectional study recruited 527 Chinese residents using a convenience sampling between December 2023 and April 2024. Inclusion criteria for residents were (1) the age of ≥18 years and (2) Chinese citizenship. Exclusion criteria for residents comprised (1) individuals with mental illnesses or those unable to communicate and (2) a prior confirmed diagnosis of dementia, stroke, Parkinson’s disease, or other conditions impacting cognitive function. The sample size was determined using Kendall’s sample size estimation method (Stuart and Ord, 2010), which suggests that the sample size should be 5 to 10 times the number of variables. This study analyzed 26 variables, including 10 items from a self-made questionnaire and 16 items from two scales. Considering a 30% inefficiency rate of the questionnaire, the minimal sample size for this study was determined at 507, the final sample size was 527.

### Data collection

Data were collected by nursing undergraduates who underwent standardized training. During their spare time, they collected data in their local communities through face-to-face interviews, during which residents were guided to independently complete the self-administered questionnaires. Before distributing the questionnaires, instructions for this study and informed consent forms were provided to the residents. Data collection commenced only after participants indicated their consent by selecting “yes.”

Regarding demographic data, all residents provided their general data, including age, gender, height, weight, place of residence, education level, marital status, household composition, personal income, and occupation.

### The ascertain dementia 8-item informant questionnaire

The self-reported AD8 has been shown to be effective in differentiating individuals with MCI from those without dementia, with its diagnostic performance also validated in studies primarily involving Chinese populations (Chin et al., 2013; Passler et al., 2021). And it created by the Alzheimer’s Disease Research Center at Washington University in 2005, is an 8-item questionnaire based on informant responses, designed to detect changes within the individual in areas such as memory, orientation, judgment, and functional abilities (Galvin et al., 2005), which may coincide with MCI, in this study, a cut-off score of two or greater suggests that the individual may have MCI (Tanwani et al., 2023; Yin et al., 2020). The informant-rated AD8 demonstrated good internal consistency, with Cronbach’s *α* = 0.84–0.85 for the English version (Galvin et al., 2006; Shaik et al., 2016) and 0.78 for the Chinese version (Li et al., 2012). It also shows strong interrater reliability, with an intraclass correlation coefficient of 0.85 for the English version (Shaik et al., 2016), and solid test–retest reliability, with weighted *κ* values between 0.67 and 0.80 for the English version (Galvin et al., 2006; Shaik et al., 2016) and an intraclass correlation coefficient of 0.96 for the Chinese version (Li et al., 2012). The AD8 is less influenced by the individual’s educational background compared to other cognitive assessment tools such as the Mini-Mental State Examination and Montreal Cognitive Assessment (Chin et al., 2013). This feature makes the AD8 particularly useful for detecting cognitive changes across diverse populations without the confounding effect of educational attainment.

### The flourishing scale

Flourishing scale (FS) is a brief 8-item summary measure of the respondent’s self-perceived success in areas such as relationships, self-esteem, purpose, and optimism (Diener et al., 2010). Each item is rated by respondents on a 7-point Likert scale (1 indicates “strongly disagree” and 7 indicates “strongly agree”), with high scores indicating high flourishing, and the total score ranging from 7 to 56 (Cerezo et al., 2024). In this study, we utilized the simplified Chinese version of the FS introduced by Tang et al. (2016), the scale shows excellent reliability and validity, with Cronbach’s *α* = 0.90–0.93 (Keyes, 2002). And Xiao et al. (2021) further classified this scale into different levels, categorizing FS scores as follows: ≥5 points represented a high flourishing level, 4–4.99 was a medium flourishing level, and < 4 denoted a low flourishing level. The exploratory factor analysis identified a single factor that accounted for 75.03% of the total variance, and the confirmatory factor analysis indicated that all the goodness-of-fit indices were acceptable (Zhang, 2018).

### Statistical analysis

All statistical analyses were conducted using Statistical Product and Service Solutions version 25.0 software (IBM, Armonk, NY, United States). Variables showing statistical differences in the univariate analysis were subsequently included in a multivariable logistic regression model to assess their net effects on cognitive function. Odds ratios (ORs) along with their 95% confidence intervals (CIs) were utilized to evaluate the independent impact of prognostic factors. The Chi-square test was used to assess the differences between groups with categorical variables. Kendall’s correlation analysis was employed to identify associations of the Ascertain Dementia 8-Item Informant Questionnaire score with general information. All *p*-values were two-tailed, with *p* < 0.05 indicating statistical significance.

### Quality control

The study was conducted anonymously to ensure the authenticity and validity of the data. Before the survey, the data collectors were trained uniformly and followed by a test after the training to ensure the accuracy and reliability of data collection. The data collectors, who were third-year nursing undergraduates from various locations, conducted the surveys in their communities therefore to reduce language barriers. Data were collected in real-time during the face-to-face interviews.

## Results

The sample included 215 males and 312 females aged 18–94 years (average: 53.36 ± 18.06 years). The healthy group comprised 356 subjects aged 18–88 years (average: 49.93 ± 17.75 years), whereas the MCI group included 170 individuals. The Kendall sample estimation algorithm was used, which estimates the sample size 10–15 times the number of variables.

### Comparing the healthy group and the MCI Group

A total of 527 participants were included in the cross-sectional study. The demographic characteristics of the residents are presented in Table 1. Based on the total AD8 scores, participants were grouped into two categories: the healthy group (*n* = 356) and the MCI group (*n* = 171), with those scoring ≥2 points on the AD8 classified as the MCI group. Compared with the healthy group, the MCI group had a higher proportion of older and overweight individuals (*p* < 0.05) but lower numbers of married, single, urban and high-income individuals, and lower FS scores (*p* < 0.05). However, no statistically significant differences were observed in gender, region, household composition, or occupation (*p* > 0.05).

**Table 1:** Demographic characteristics of the residents.

Variables	Group	Total *n* (%)	Health *n* (%)	Mild cognitive impairment *n* (%)	*χ^2^*	*p*
Gender	Male	215 (40.80%)	154 (43.30%)	61 (35.7%)	2.752	0.097
	Female	312 (59.20%)	202 (56.70%)	110 (64.3%)		
Age	≤59	319 (60.50%)	247 (69.40%)	72 (42.10%)	36.718	<0.001
	60–69	95 (18.00%)	52 (14.60%)	43 (25.10%)		
	70–79	91 (17.30%)	47 (13.20%)	44 (25.70%)		
	≥80	22 (4.20%)	10 (2.80%)	12 (7.00%)		
Region	Inside Sichuan	416 (78.90%)	277 (77.80%)	139 (81.30%)	0.84	0.359
	Outside Sichuan	111 (21.10%)	79 (22.20%)	32 (18.70%)		
Marital status	Married	379 (71.90%)	264 (74.20%)	116 (67.30%)	36.447	<0.001
	Divorced	11 (2.10%)	6 (1.70%)	5 (2.90%)		
	Widowed	55 (10.40%)	19 (5.30%)	35 (21.10%)		
	single	82 (15.60%)	67 (18.80%)	15 (8.80%)		
Household composition	Living alone	45 (8.50%)	25 (7.00%)	20 (11.70%)	3.696	0.158
	Living with spouse	209 (39.70%)	147 (41.30%)	62 (36.30%)		
	Living with children or other family members	273 (51.80%)	184 (51.70%)	89 (52.00%)		
Occupation^a^	None	132 (25.00%)	87 (24.40%)	45 (26.30%)	0.369	0.832
	Mental work	83 (15.70%)	58 (16.30%)	25 (14.60%)		
	Physical work	312 (59.20%)	211 (59.30%)	102 (59.10%)		
Education level	Primary school and below	233 (44.20%)	135 (37.90%)	98 (57.30%)	21.631	<0.001
	Junior high school	125 (23.70%)	86 (24.20%)	39 (22.80%)		
	High school and above	169 (32.10%)	135 (37.90%)	34 (19.90%)		
Personal income (monthly)	Less than 2000 RMB	268 (50.90%)	158 (44.40%)	110 (64.30%)	18.387	<0.001
	More than 2000 RMB	259 (49.10%)	198 (55.60%)	61 (35.70%)		
BMI	Underweight	45 (8.50%)	32 (9.00%)	13 (7.60%)	7.973	0.047
	Normal	361 (68.50%)	246 (69.10%)	115 (67.30%)		
	Overweight	101 (19.20%)	60 (16.90%)	41 (24.00%)		
	Obese	20 (3.80%)	18 (5.10%)	2 (1.20%)		
FS level	Low level	60 (11.40%)	29 (8.10%)	31 (18.10%)	31.992	<0.001
	Medium level	140 (26.60%)	77 (21.60%)	63 (36.80%)		
	High level	327 (62.00%)	250 (70.20%)	77 (45.30%)		
Place of residence	Urban	345 (65.50%)	277 (77.80%)	68 (39.80%)	73.946	<0.001
	Rural	182 (34.50%)	79 (22.20%)	103 (60.20%)		

a Using the Occupational Classification of the People’s Republic of China (2015 edition) as a reference, we categorized occupations into two groups: mental work (e.g., professionals, government employees) and physical work (e.g., farmers, factory workers).

FS, flourishing scale.

### Correlation analysis of MCI

Kendall’s correlation analysis showed a negative correlation between FS (*b* = −0.237, *p* < 0.001), education level (*b* = −0.187, *p* < 0.001), personal income (*b* = −0.191, *p* < 0.001), and the AD8 score. Additionally, age (*b* = 0.248, *p* < 0.001) was positively correlated with the AD8 score (Table 2). The factor values are listed in Table 3.

**Table 2:** Kendall’s correlation analysis of Mild Cognitive Impairment with general information.

	FS level	age	Education level	Personal income
AD8 score				
*b*	−0.237	0.248	−0.187	−0.191
*p* value	p < 0.001	p < 0.001	p < 0.001	p < 0.001

FS, flourishing scale.

**Table 3:** Factor values of the independent variables.

Values	Value
FS level	1 = Low level, 2 = Medium level, 3 = High level
age	1 = ≤59, 2 = 60 ~ 69, 3 = 70 ~ 79, 4 = ≥80
Education level	1 = Primary school and below, 2 = Junior high school, 3 = High school and above
Personal income (monthly)	1 = Less than 2000 RMB, 2 = More than 2000 RMB
Marital status	1 = Married, 2 = Divorced, 3 = Widowed, 4 = single
BMI	1 = Underweight, 2 = Normal, 3 = Overweight, 4 = Obese
Place of residence	1 = Urban, 2 = Rural

Univariate logistic regression analysis revealed a statistically significant difference in age, place of residence, and FS level. Age [OR = 1.451, 95% CI (1.107–1.902), *p* = 0.007], FS [OR = 0.421, 95% CI (0.311–0.569), *p* < 0.001], and place of residence [OR = 5.523, 95% CI (3.572–8.539), *p* < 0.001] continued to be independently linked with MCI (Table 4).

**Table 4:** Multivariate logistic regression analyses of factors affecting cognitive dysfunction.

	Multivariate analysis		
	*OR*	95% CI	*p* value
Age	1.451	1.107–1.902	0.007
Education level	0.784	0.574–1.069	0.124
Personal income (monthly)	0.625	0.387–1.008	0.054
FS level	0.421	0.311–0.569	<0.001
Marital status	1.011	0.818–1.25	0.918
BMI	1.151	0.811–1.634	0.431
Place of residence	5.523	3.572–8.539	<0.001

FS, flourishing scale.

## Discussion

### Influencing factors of MCI

Numerous conditions can cause a decline in cognitive function and dementia. This study indicated that MCI is associated with age, personal income, and especially FS scores.

#### Age and rural residency are the influencing factors

The risk of MCI increases with age and among rural residents, of which age is a nonmodifiable factor (Jia et al., 2020a; Alzheimer's Association, 2015). Age-related MCI is multifactorial, with numerous underlying and frequently co-morbid pathological correlates (Mckenzie et al., 2022). Research indicated that various forms of brain pathology (such as cerebrovascular disease, neuritic plaques, neurofibrillary tangles, Lewy body disease, TDP-43 pathology, and hippocampal sclerosis) can be linked with an increased risk of age-related mild cognitive impairment (Kapasi et al., 2017; Power et al., 2018). Furthermore, living in rural areas is a major risk factor for mild cognitive impairment (Vega and Newhouse, 2014). A study among older Indians demonstrated significant differences in cognitive impairment based on urban or rural residential status, with rural residence identified as a significant risk factor for cognitive impairment (Muhammad, 2023). Similarly, a cross-sectional survey of residents aged ≥65 years from 30 urban and 45 rural communities across China revealed that the prevalence of dementia and AD was notably higher in rural areas than in urban areas (Jia et al., 2014). Urban areas provide better transportation, healthcare access, health information, infrastructure, and educational opportunities, all of which contribute to improved health and quality of life for older residents. In contrast, older adults in rural areas face limited and less adequate services in these areas, increasing the likelihood of developing MCI.

#### Flourishing is an important factor related to MCI

Our study found that flourishing is significantly associated with a lower risk of MCI. Specifically, participants with higher flourishing level had a reduced likelihood of MCI (OR = 0.421, 95%CI: 0.311—0.569, *p* < 0.001). This finding suggest that flourishing may have a protective effect on cognitive function, potentially by enhancing psychological resilience. Consistent with prior research, our findings indicated that flourishing may protect against cognitive decline by fostering psychological resilience. Psychosocial resilience factors, such as positive emotional states and social support, may contribute more significantly to cognitive well-being than physical health alone (Reichstadt et al., 2007; Jeste et al., 2013; O'brien et al., 2023). Furthermore, psychological resilience and competence play a significant role in slowing down biological aging (Zábó et al., 2023), which aligns with our observation that individuals with higher flourishing scores exhibited better cognitive performance. These may help individuals more effectively cope with neuropathological changes, potentially delaying cognitive decline.

### Correlation between flourishing and MCI

This study identified a correlation between flourishing and MCI. It indicated that individuals with higher flourishing levels have a significantly lower incidence of MCI, with a correlation coefficient of −0.237 (*p* < 0.001). This suggests that flourishing plays a meaningful role in reducing the risk of MCI and may serve as a protective factor in maintaining cognitive function. However, the research focusing on the relationship between MCI risk and flourishing is scarce. A randomized controlled trial involving 51 participants suggested that positive psychology interventions can enhance the subjective well-being of individuals diagnosed with amnestic MCI, with the benefits persisting for some time after the intervention ends (Tsiflikioti et al., 2023). The study also reflected that MCI may be related to the relatively intact emotional function in early AD (Bozeat et al., 2000). Flourishing may protect cognitive function through multiple pathways, including reducing stress-related neurotoxicity and enhancing emotional regulation. Chronic stress is known to elevate cortisol levels, which can accelerate hippocampal atrophy—a key brain region implicated in early AD (Wells et al., 2019; Hakeem et al., 2025; Sharan and Vellapandian, 2024). However, individuals with higher flourishing levels tend to exhibit greater psychological resilience, which has been linked to lower cortisol reactivity (Heller et al., 2013) and lower stress (Laakso et al., 2025). Additionally, flourishing fosters positive emotional states, which are associated with increased dopamine (Chopra, 2023) and serotonergic activity (Fan et al., 2023), both of which play crucial roles in cognitive processing and emotional regulation (Echouffo-Tcheugui et al., 2018; Ott and Nieder, 2019; Švob Štrac et al., 2016). By mitigating chronic stress and promoting emotional stability, flourishing may serve as a buffer against MCI risk and cognitive decline.

Chronic stress is a well-established risk factor for MCI and AD, primarily due to its impact on the hippocampus, a brain region highly susceptible to stress-induced atrophy (Kennedy et al., 2017; Hyer et al., 2021). Elevated stress levels lead to dysregulation of the hypothalamic–pituitary–adrenal (HPA) axis, resulting in prolonged cortisol exposure, which in turn accelerates neuronal damage and cognitive decline (Magri et al., 2006; Tsigos and Chrousos, 2002). However, increasing evidence suggests that positive psychology techniques, particularly those that foster flourishing, can effectively enhance subjective well-being and mitigate stress levels (Machado et al., 2019; Kinoshita et al., 2024). Flourishing has been linked to improved emotional regulation and lower cortisol reactivity (De Vries et al., 2022), potentially protecting the hippocampus from stress-induced damage. Therefore, flourishing may act as a protective factor against MCI, reducing the impact of stress and emotional disturbances on cognitive health.

Conclusively, in the future, greater attention should be given to the psychological aspects of patients with MCI. Instead of focusing solely on the negative aspects, more positive psychological interventions, such as psychological counseling, positive psychology training, and social support programs, should be implemented. These interventions can improve individuals’ flourishing levels, thereby enhancing their cognitive function.

### Limitations

This study has some limitations, including the use of a non-random sampling method due to constraints in time and workforce, which may have affected generalizability. Future research should improve sampling methods and adopt longitudinal designs to explore causal relationships between flourishing and MCI. Additionally, future studies need to investigate specific impacts of flourishing on various types of MCI.

## Data Availability

The raw data supporting the conclusions of this article will be made available by the authors, without undue reservation.
